# Supplementary material for: Impact of SARS-CoV-2 on healthcare and essential workers: A longitudinal study of PROMIS-29 outcomes
Source: PLoS One. 2025 Jul 18;20(7):e0324755. doi: 10.1371/journal.pone.0324755 (PMC12273909; doi:10.1371/journal.pone.0324755)
Supplement: S1 Appendix — Appendix 1: Acknowledgements INSPIRE Group Author List. Appendix 2: Demonstration of Adjusted Effect Estimation from GEE Modeling. (DOCX) [file pone.0324755.s001.docx]

**Appendix 1: ACKNOWLEDGEMENTS INSPIRE Group Author List**

**Rush University, Administrative Core & Enrolling Site**

**Study-wide Co- Principal Investigators:** Robert A. Weinstein, MD, Principal Investigator; Michael Gottlieb, MD, Principal Investigator

**Core research team:** Michelle Santangelo, MS, Research Manager; Katherine Koo, MS-HSM, Program Manager; Antonia Derden, BA, Administrative Assistant

**Site Investigators:** Michael Gottlieb, MD, Site Principal Investigator

**Site research team:** Kristyn Gatling, MA, Research Coordinator. Research Assistants: Zohaib Ahmed, MS; Chloe Gomez; Diego Guzman, BS; Minna Hassaballa, BA; Ryan Jerger; Amro (Marshall) Kaadan, ScM

**Yale University, Analytic Core & Enrolling Site**

**Core Investigators:** Principal Investigators: Arjun Venkatesh, MD, MBA, MHS; Erica S. Spatz MD, MHS

**Core research team:** Research Managers: Jeremiah Kinsman, MPH, NREMT, Caitlin Malicki**,** MPH. Statisticians: Zhenqiu Lin, PhD; Shu-Xia Li, PhD; Huihui Yu, PhD; Imtiaz Ebna Mannan, MS; Zimo Yang, MS; Mengni Liu, MS

**Site Investigators:** Site Principal Investigators: Arjun Venkatesh, MD, MBA, MHS, Erica S. Spatz MD, MPH. Site Co-Investigator: Andrew Ulrich, MD

**Site Research team:** Research Managers: Jeremiah Kinsman, MPH, NREMT, Caitlin Malicki, MPH. Research Coordinator: Jocelyn Dorney, MPH. Research Assistants: Senyte Pierce, BA; Xavier Puente, BA; Wafa Salah, BA

**University of Washington, Clinical Core & Enrolling Site**

**Core Investigators:** Graham Nichol, MD, Principal Investigator; Kari A. Stephens PhD, MS, Co-Principal Investigator

**Core research team:** Jill Anderson, BSN, RN, Clinical Core Program Manager; Mary Schiffgens, MBA, Grant & Finance Manager; Dana Morse, RN, BSN, Research Coordinator; Karen Adams, BA, Regulatory Specialist; Tracy Stober, BA, MA, Patient Representative; Zenoura Maat, Research Assistant

**Site Investigators:** Kelli N. O’Laughlin, MD, MPH, Site Principal Investigator; Nikki Gentile, MD, PhD, Co-Investigator

**Site research team:** Research Coordinators: Rachel E. Geyer, MPH; Michael Willis, AS, BSHS; Zihan Zhang, MS, Analyst; Gary Chang, PhD, Senior Biostatistician. Victoria Lyon, MPH, Project Manager. Research Assistants: Robin E. Klabbers, MSc in Medicine, MSc in Global Health; Luis Ruiz, BA; Kerry Malone, BA; Jasmine Park

**Thomas Jefferson University, Enrolling Site**

**Site Investigators:** Kristin Rising, MD, MS, Site Principal Investigator; Efrat Kean, MD, Co-Investigator; Anna Marie Chang, MD, MSCE

**Site research team:** Nurse Coordinator: Nicole Renzi, RN. Program Manager: Phillip Watts, BA, MM, CCRP. Research Coordinators: Morgan Kelly, BS; Kevin Schaeffer, BS; Dylan Grau, BS; David Cheng, BS; Carly Shutty, BSN; Alex Charlton, BS; Lindsey Shughart, BS; Hailey Shughart, BA, CCRP; Grace Amadio, MD, CCRP; Jessica Miao, BA. Research Assistants: Paavali Hannikainen, BS

**University of California, Los Angeles, Enrolling Site**

**Site Investigators:** Joann G. Elmore, MD, MPH, Site Principal Investigator, Lauren E. Wisk, PhD, Co-Investigator

**Site research team:** Michelle L’Hommedieu, PhD, Site Program Director; Chris Chandler, BA, Research Assistant; Megan Eguchi, MPH, Data Analyst; Kate Diaz Roldan, MPH, Research Assistant; Raul Moreno, BA, Administrative Analyst

**University of California, San Francisco, Enrolling Site**

**Site Investigators:** Robert Rodriguez, MD, Site Principal Investigator; Ralph C. Wang, MD, MAS, Site Principal Investigator; Juan Carlos Montoy, MD, PhD, Site Principal Investigator

**Site research team:** Robin Kemball, MPH, Program Manager; Research Coordinators: Virginia Chan, MPH; Cecilia Lara Chavez; Angela Wong, BA; Mireya Arreguin, BS

**University of Texas Health Science Center at Houston, Enrolling Site**

**Site Investigators:** Mandy J. Hill, DrPH, MPH, Site Principal Investigator; Ryan Huebinger Site, MD, Site Principal Investigator.

**Site research team**: Arun Kane, BA, Research Coordinator; Peter Nikonowicz, BA, Research Coordinator; Sarah Sapp, MPH, Research Coordinator

**University of Texas Southwestern Medical Center, Enrolling Site**

**Site Investigators:** Ahamed H. Idris, MD, Site Principal Investigator; Samuel McDonald, MD, Co-Investigator

**Site research team:** David Gallegos, BS, Research Coordinator; Katherine Riley Martin, BS, MS, Research Assistant

**Centers for Disease Control and Prevention (CDC)**

**Investigators:** Sharon Saydah, PhD; Ian D. Plumb, MBBS, MSc; Aron J. Hall, DVM, MSPH; Melissa Briggs-Hagen, MD, MPH

**Public Health Seattle King County:** We would like to thank Public Health Seattle King County for their assistance with participant recruitment for this study.

**California Department of Public Health**: We would like to thank the California Department of Public Health for their assistance with participant recruitment for this study.

**CTSI COVID Clinical Research Steering Committee and the CTSI Office of Clinical Research Patient Navigation Team and Bioinformatics Program**: We would like to thank the CTSI COVID Clinical Research Steering Committee and the CTSI Office of Clinical Research Patient Navigation Team and Bioinformatics Program for assistance with study recruitment.

**University of Washington Institute of Translational Health Sciences (ITHS):** We would like to thank the ITHS for support of the REDCap instance and for biomedical informatics resources used by the UW Clinical Core and Enrolling Site to enable study recruitment, which is funded by the National Center for Advancing Translational Sciences of the National Institutes of Health under award number UL1TR002319.

| **Appendix 2. Demonstration of Adjusted Effect Estimation from GEE Modeling**  Example of calculating the marginal worker group effects on each of the 4 continuous outcomes (e.g., PROMIS29 physical health summary) at a time point:  In the GEE models using the identity link, the mean function for an outcome for patient $i$ at survey time $t$ *,* $\mu_{it}$is estimated as below.  $logit\left( \mu_{it} \right)=\beta_{0}+{covid}_{i}*\beta_{1}+{threemonth}_{it}*\beta_{2}+{longterm}_{it}*\beta_{3}+{healthcare}_{i}*\beta_{4}+{essential}_{i}*\beta_{5}+\left( covid_{i}*{threemonth}_{it} \right)*\beta_{6}+ \left( covid_{i}*{longterm}_{it} \right)*\beta_{7}+ \left( covid_{i}*{healthcare}_{i} \right)*\beta_{8}+\left( covid_{i}*{essential}_{i} \right)*\beta_{9}+\left( {threemonth}_{it}*{healthcare}_{i} \right)*\beta_{10}+\left( {threemonth}_{it}*{essential}_{i} \right)*\beta_{11}+\left( {longterm}_{it}*{healthcare}_{i} \right)*\beta_{12}+\left( {longterm}_{it}*{essential}_{i} \right)*\beta_{13}+ {\boldsymbol{X}_{\boldsymbol{i}}}^{\boldsymbol{'}}*\boldsymbol{B}$  Where we have included interactions between 1) worker groups and COVID-19 status, 2) worker groups and survey time points, and 3) index COVID status and survey time pints; $\boldsymbol{X}_{\boldsymbol{i}}$ denotes a vector of observed values of 8 time-invariant risk variables for patient $i$, $\boldsymbol{B}$ denotes these covariates’ coefficients for outcome (e.g., PROMIS29 physical health summary), and  ${covid}_{it}=\left\{ \begin{aligned} 1, &if COVID+ \\ 0, &else \end{aligned} \right.$ ;  ${threemonth}_{i}=\left\{ \begin{aligned} 1, &if survey taken at 3-month \\ 0, &else \end{aligned} \right.$ ;  ${longterm}_{i}=\left\{ \begin{aligned} 1, &if survey taken at the latest timepoint among 12, 15, and 18 months \\ 0, &else \end{aligned} \right.$ ;  ${healthcare}_{it}=\left\{ \begin{aligned} 1, &if the worker group is healthcare \\ 0, &else \end{aligned} \right.$  ${essential}_{it}=\left\{ \begin{aligned} 1, &if the worker group is non-healthcare essential \\ 0, &else \end{aligned} \right.$ ;  The estimated coefficients from the GEE modeling are used to calculate the marginal effect. For example, the marginal effect of a covid-positive healthcare worker’s average physical summary score at 3-month in comparison to a covid-positive general worker’s score at 3-month is calculated as below:  ${\mu_{physical\_summary}\left( healthcare vs. general \right)\vert}_{3month, COVID+}=exp\{\hat{\beta}_{4}+ \hat{\beta}_{8}+\hat{\beta}_{10}\}$ |
| --- |
