# Supplementary material for: Impact of SARS-CoV-2 on healthcare and essential workers: A longitudinal study of PROMIS-29 outcomes
Source: PLoS One. 2025 Jul 18;20(7):e0324755. doi: 10.1371/journal.pone.0324755 (PMC12273909; doi:10.1371/journal.pone.0324755)
Supplement: Attachment — Submitted filename: Response to Reviewers_3-5-25.docx [file pone.0324755.s003.docx]

Response to Reviewers

**Thank you for the opportunity to revise this manuscript. We appreciate the Editors and Reviewers comments and have addressed them below and in the manuscript.**

Reviewer #1: This is an important article to address the patient-reported outcomes among essential workers compared with the general working population following SARS-CoV-2 infection and in the context of the COVID-19 pandemic. I recommended acceptance for publication. The only interested further analysis is whether the patient-reported outcomes are different among different SARS-CoV2 variants.

**Thank you for your comments and recommendation that this manuscript be accepted for publication. Your recommendation for further analysis is warranted and noted.**

Reviewer #2: A review of the manuscript entitled “Impact of SARS-CoV-2 on Healthcare and Essential Workers: A Longitudinal Study of PROMIS-29 Outcomes”

1. The authors have clearly written this manuscript in professional, unambiguous language. I appreciate the authors for their extensive data set, which included participants from eight US sites. However, there are some minor things that need to be rectified to increase the quality of this study.

**Thank you for your comments and suggestions.**

2. (page 16) please revise the study timing from this sentence using a unified format: “A total of 8950 participants enrolled in the study between 12/7/2020 and 8/29/22, of which 6,044 were eligible for follow-up based on the three sets of eligibility criteria above.”

**This has been corrected to state: “A total of 8,950 participants enrolled in the study between 12/7/2020 and 8/29/2022, of which 6,044 were eligible for follow-up based on the three sets of eligibility criteria above.”**

3. Authors are suggested to proofread the manuscript after addressing all comments to avoid any typological, grammatical, and lingual mistakes and errors. For example, “Each site began their enrollment” on page 16;

**Thank you for noticing this error, this clause has been deleted.**

“…after (omicron) this period.”

**Thank you for highlighting this error, it was corrected to state “19% enrolled during the omicron variant period”**

on page 20; “Among essential non-HCWs…” on page 24.

**Thank you for highlighting this grammatical error, among has been changed to “For”**

4. Before delving further into COVID-19 in the US, I suggest that authors elaborate more on what COVID-19 itself is first. For example, “The COVID-19 pandemic has profoundly impacted millions of lives worldwide. By June 2023, the global count of confirmed COVID-19 cases exceeded 767 million, with over 6.9 million fatalities. Healthcare workers have borne the brunt of the pandemic's effects, with the World Health Organization (WHO) reporting that over 14% of COVID-19 cases involve this group. The rising incidence of COVID-19 among healthcare professionals risks significantly straining healthcare systems, reducing service quality, and leading to adverse health outcomes.” This information can be cited from “Fatigue in healthcare workers with mild COVID-19 survivors in Indonesia”.

**This reference has been added and the suggested text was condensed to: “The COVID-19 pandemic has profoundly impacted millions of lives worldwide. By June 2023, the global count of confirmed COVID-19 cases exceeded 767 million, with over 6.9 million fatalities. Healthcare workers have borne the brunt of the pandemic's effects, with the World Health Organization (WHO) reporting that over 14% of COVID-19 cases involve this group [1].”**

5. (page 14, introduction) This sentence “Frontline workers risked their personal and household’s health and well-being, with ongoing exposure to severe acute respiratory syndrome coronavirus 2 (SARS-CoV-2)…” also shares the same idea with a similar study by Hamdan et al. entitled “Coping strategies used by healthcare professionals during COVID-19 pandemic in Dubai: A descriptive cross-sectional study”. Kindly include this reference to make the sentence stronger.

**Thank you for sharing more supporting information, this paper has been added as reference 5 in the manuscript.**

6. (page 15, methods) I suggest that authors arrange the information on the start dates for each institution in a table to make it more organised.

**Thank you for your suggestion to increase organization, I have created a new table 1 to organize this information appropriately.**

**Table 1: Institutions start date of enrollment in the INSPIRE Study**

| Institution | Start Date |
| --- | --- |
| Rush University | November 17, 2020 |
| University of Washington | December 11, 2020 |
| Yale University | December 26, 2020 |
| University of California, Los Angeles | February 1, 2021 |
| Thomas Jefferson University | February 8, 2021 |
| University of California, San Francisco (UCSF) | February 24, 2021 |
| University of Texas Southwestern (UTSW) | April 21, 2021 |
| University of Texas Health Science Center at Houston (UTH) | May 5, 2021 |

Reviewer #3: Dear Authors,
Thank you for this article. It is an exciting article to publish. The title is Impact of SARS-CoV-2 on Healthcare and Essential Workers: A Longitudinal Study of PROMIS-29 Outcomes, and the ID number is (PONE-D-24-50826). The findings can be compared to general workers; essential HCW status had higher cognitive function in the absence of SARS-CoV-2 infection at all time points, while essential non-HCWs were most vulnerable to poor recovery in long-term physical health following SARS-CoV-2 infection. Preparation efforts for future pandemics may consider enhanced protection and post-infection resources for frontline workers.
- The results in this article are good.
Finally, this article is acceptable for publishing because it is the new result and well written.

**Thank you for your comments and recommendation for this manuscript to be published.**
